# Motivation Counts: Autonomous But Not Obligated Sharing Promotes Happiness in Preschoolers

**DOI:** 10.3389/fpsyg.2017.00867

**Published:** 2017-05-31

**Authors:** Zhen Wu, Zhen Zhang, Rui Guo, Julie Gros-Louis

**Affiliations:** ^1^Department of Psychology, Tsinghua UniversityBeijing, China; ^2^Key Laboratory of Behavioral Science, Institute of Psychology, Chinese Academy of SciencesBeijing, China; ^3^Department of Psychology, University of Chinese Academy of SciencesBeijing, China; ^4^Department of Psychological and Brain Sciences, University of Iowa, Iowa CityIA, United States

**Keywords:** sharing, happiness, prosocial behavior, social norm, preschooler

## Abstract

Research has demonstrated that prosocial sharing is emotionally rewarding, which leads to further prosocial actions; such a positive feedback loop suggests a proximal mechanism of human’s tendency to act prosocially. However, it leaves open a question as to how the emotional benefits from sharing develop in young children and whether sharing under pressure promotes happiness as well. The current study directly compared 3- and 5-year-old Chinese children’s happiness when sharing was autonomous (the recipient did not contribute to getting the reward) with when sharing was obligated (the recipient and the actor jointly earned the reward). We found that children shared more items overall when sharing was obligated than autonomous, demonstrating their conformity to social norms of merit-based sharing. In children who eventually shared with others, 5-year-olds gave out more stickers in the obligated sharing condition than in the autonomous sharing condition, but 3-year-olds shared the same amount between the conditions, suggesting that 5-year-olds adhered to the merit-based sharing norm more strictly than 3-year-olds. Moreover, in the autonomous sharing condition, children displayed greater happiness when they shared with the recipient than when they kept stickers for themselves, suggesting that costly prosocial giving benefited children with positive mood; however, children did not gain happiness when they shared with the recipient in the obligated sharing condition. These findings demonstrate that children’s affective benefits depend on the motivation underlying their prosocial behavior, and further imply that normative force and emotional gains may independently drive preschoolers’ prosocial behaviors.

## Introduction

Traditional economic theory posits that human beings are rational, and are motivated by self-interest. However, contrary to this perspective, humans routinely show prosocial behaviors that require them to incur personal costs to benefit others, such as donating, engaging in charitable activities, and saving strangers’ lives. This puzzle has fascinated social scientists for decades: what inspires and motivates people to perform prosocial behaviors even when faced with loss of resources and potential physical harm (for a debate see Decety and Svetlova, 2012; Dunfield and Kuhlmeier, 2013; Dunfield, 2014; Paulus, 2014; Carpendale et al., 2015; Martin and Olson, 2015)?

One intriguing claim is that an affective self-reward mechanism helps people maintain prosocial behavior (Dunn et al., 2014). That is, engaging in prosocial actions promotes positive mood, which in turn leads to further prosocial behavior; such a positive-feedback loop offers a path to sustain prosocial behavior (Aknin et al., 2011a). In support of this hypothesis, recent studies with adults have shown that spending money on others leads to greater happiness than spending money on themselves (Dunn et al., 2008). Furthermore, spending money on people with strong social ties (e.g., family and friends) leads to greater happiness than spending on people with weak social ties (e.g., less frequent contact, lower emotional intensity, and limited intimacy) (Aknin et al., 2011b). This phenomenon is universal across cultures (Aknin et al., 2013a). The emotional reward is not just subjective feeling, but can be perceived by third-party observers; participants’ self-reported emotion ratings after prosocial spending are correlated to observers’ coding of the participants’ emotions (Aknin et al., 2014). In addition, charity donations activate regions of the brain related to reward processing, which is proposed to support the existence of the self-reward mechanism (Harbaugh et al., 2007).

Children have also been shown to glean affective benefits from acting prosocially. For example, 22-month-old toddlers exhibited greater happiness when giving their own treats to a puppet than when receiving treats themselves or giving an experimenter’s treats to the puppet, suggesting that costly giving rewards young children with positive emotions (Aknin et al., 2012). There is also a cognitive basis of the relation between generosity and happiness in young children. Paulus and Moore (2016) recently demonstrated that 3–6 year old children expected people to be happier after sharing than after not sharing, and the individual differences in their understanding of the relation between sharing and their own emotions predicted generosity in a subsequent sharing task. Moreover, encouraging children aged 9–11 to engage in charitable activities can effectively improve children’s well-being (Layous et al., 2012). These findings provide evidence that prosocial actions reward children with positive feelings, which may partly explain instances of generosity in children.

The existing studies on the relationship between prosocial behavior and positive emotions have only focused on altruistic and voluntary sharing; however, there are strong social norms that influence children’s sharing behavior as well. Thus, an open question is whether people gain happiness when pressured to share by social norms. One prosocial norm is sharing according to merit: one should distribute rewards according to how much someone contributes to a task. Studies have shown that children as young as 3 years old take merit into account by sharing resources according to individual contribution (Warneken et al., 2011; Baumard et al., 2012; Kanngiesser and Warneken, 2012; Hamann et al., 2014; Schmidt et al., 2016). Three-year-olds also spontaneously protest against unfair distributions if the actor and the recipient jointly earned the resources, demonstrating their understanding of the merit-based norm of resource distribution (Rakoczy et al., 2016). This understanding of merit-based sharing increases with age (Schmidt et al., 2016). Despite the great interest in studying children’s understanding of, and compliance with, the normative force of sharing, it remains a question whether children gain happiness when their sharing is obligated according to social norms as much as when they share autonomously. Do children experience happiness from following a merit-based sharing norm as they do from sharing voluntarily and altruistically?

Investigating this issue is important for understanding the mechanisms of the emergence and development of prosocial behaviors. As reviewed above, in the literature, the aforementioned two possible mechanisms – a prosocial orientation is motivated by the anticipation of positive affect (“pleasure-based”), or to fulfill a duty or conform to a social norm (“pressure-based”) – have been examined separately in two lines of research. It is crucial to distinguish between these two kinds of prosocial motivations because the “pleasure-based” motivation for prosociality directly impacts individuals’ affective experience, thus it may have more impact on positive outcomes. For instance, a survey study showed that the pleasure-based prosocial orientation was positively correlated to adults’ subjective well-being, but the pressure-based prosocial motivation was not (Gebauer et al., 2008). In addition, adults became happier when helping was autonomous (they could choose freely to help others) than when helping was controlled (Weinstein and Ryan, 2010). Despite the findings from adult populations, little is known about whether young children derive differential happiness from autonomous sharing versus obligated sharing. When there is a social norm of merit-based sharing to obey, children as young as three can follow the social norm, suggesting that they may view sharing as an obligation in this situation. Does the underlying motivation for exhibiting prosocial behavior differentially influence children’s subjective well-being?

To answer these questions, in the current study, we directly compared children’s affective benefits of sharing with a recipient in two conditions: the recipient did not make contributions to earning a reward received by the child participant (an autonomous sharing condition), or the recipient completed half of the work for the reward received by the participant (an obligated sharing condition). In both conditions, children could decide alone whether they wanted to share the reward and, if so, how much they wanted to share. It was expected that if children conform to social norms of merit-based sharing, they would share more in the obligated sharing condition. The primary question we aimed to address is whether children gain happiness when they are obligated to share as has been shown previously in situations when people share altruistically and voluntarily. Answering this question can further our understanding of the mechanisms underlying children’s prosocial behaviors. As reviewed above, previous research suggests that children as young as 22 months old gain happiness from sharing (Aknin et al., 2012); moreover, children start acting in accordance with merit-based sharing norms around 3 years of age (e.g., Rakoczy et al., 2016), and this norm understanding increases with age (e.g., Schmidt et al., 2016). Given these findings, we examined 3- and 5-year-old preschool children with the established paradigm for studying sharing behavior.

## Materials and Methods

### Participants

Participants were 139 children, including 51 3-year-olds (*M* = 41.18 months, *SD* = 3.02, range = 33–45 months; 24 boys) and 88 5-year-olds (*M* = 64.40 months, *SD* = 3.03, range = 59–70 months; 40 boys). They were assigned randomly to one of two conditions. For 3-year-olds, 27 children (*M* = 40.85 months, *SD* = 3.07, range = 33–45 months; 13 boys) were assigned to the autonomous sharing condition, and 24 children (*M* = 41.54 months, *SD* = 2.99, range = 36–45 months; 11 boys) were in the obligated sharing condition; the age difference between the two conditions was not significant, *F*(1,49) = 0.66, *p* = 0.422, Δ*η*^2^ = 0.01. For 5-year-olds, 43 children (*M* = 64.56 months, *SD* = 3.27, range = 59–70 months; 21 boys) were in the autonomous sharing condition, and 45 children (*M* = 64.24 months, *SD* = 2.81, range = 59–70 months; 19 boys) were in the obligated sharing condition; the age difference between the two conditions was not significant either, *F*(1,86) = 0.23, *p* = 0.630, Δ*η*^2^ = 0.003. All participants were typically developing Chinese children from three kindergartens in Beijing, China. This study was approved by the Institutional Review Board at the Institute of Psychology – Chinese Academy of Sciences and the Institutional Review Board at Tsinghua University, and written parental consent was provided for all subjects.

### Materials and Procedure

Each child participated in a sticker sharing task in a quiet room in their kindergarten. A female experimenter sat across a table from the child. All experimental sessions were video recorded.

In both the autonomous and obligated sharing conditions, children received six stickers as a reward for completing a puzzle, and they could decide freely whether to share with a partner. The partner was not present, and was described as an anonymous child of the same age and gender as the participant, so that the participant and the partner never actually interacted with each other. In both conditions, the partner completed half of a puzzle but did not get stickers; however, the critical difference between the two conditions was (1) whether the partner contributed to earning the reward *together* with the participant for completing a puzzle jointly; and (2) whether the stickers belonged to just the child or to both the child and the partner. Specifically, in the autonomous sharing condition, children were told that the experimenter completed half of a puzzle yesterday and children were asked to complete the other half of the puzzle. Children were also told that the partner completed a half of another puzzle (did the same thing as the child did) yesterday, but failed to get stickers because the experimenter ran out of stickers. When the child received the six stickers after completing the puzzle, s/he was told that these stickers belonged to him or her because of finishing the puzzle. Therefore, in the autonomous sharing condition, the partner did not contribute to completing the puzzle for which the child was earning stickers. Sharing was thus autonomous because the child owned those stickers, and it was not his or her obligation to share; rather, it was the experimenter’s responsibility to bring enough reward. By contrast, in the obligated sharing condition, children were told that the partner completed the other half of the child’s puzzle, but s/he did not get stickers yesterday since the puzzle was not completed. When the child received the six stickers after completing the puzzle, s/he was told that these stickers belonged to both him or her and their partner, because they both worked to finish the game. Therefore, in the obligated sharing condition, the partner and the participant jointly worked to earn the reward (though they each completed half of the work separately at different times); if children are influenced by the merit-based social norm and the claim that the stickers belonged to both of them, they should feel obligated to share because the recipient deserves the reward obtained by children.

After the child completed the puzzle and received the stickers, he or she was given two envelopes with different colors, one for himself/herself, and the other one for the absent partner. The experimenter then told the child that she would turn her back to the child so that she would not see how the child allocated stickers, and the child could decide freely how many stickers to be put into each of the two envelopes – s/he could take all of the stickers home by placing the stickers in his or her own envelope and that, should s/he wish to, s/he could share some of the stickers with the other child by placing stickers into the other envelope. This paradigm is widely used in developmental research studying children’s sharing behavior (e.g., Blake and Rand, 2010; Gummerum et al., 2010; Engelmann et al., 2013; Xiong et al., 2016), because it enables children to be free to share any portion (including all or none) of the stickers without being influenced by the presence of social partners.

A series of questions were then asked to confirm that children understood the rules, including (a) to whom these stickers were rewarded, (b) which envelope to put the stickers for themselves and the other child, and (c) whether the experimenter could see how they shared after she turned around. Only after children correctly answered all the above comprehension questions would they proceed to the sharing task. If children answered the questions correctly, the experimenter would acknowledge by saying something like “you are right! You own the stickers because you completed the other half of the puzzle today” (in the autonomous sharing condition) or “you are right! The stickers belong to both you and her/him because you each completed a half of the puzzle” (in the obligated sharing condition). If children did not successfully answer the questions the first time, the story would be repeated and explained by the experimenter. The majority of children answered questions correctly at the first time; only a few children required a repetition to pass the comprehension questions.

### Coding

The number of stickers that participants shared was recorded by counting how many stickers the child put in the recipient’s envelope. In addition, participants’ emotional expressions were coded by assistants who were kept blind to experimental hypotheses and the experimental condition that the child was in. Children’s happiness was coded on a seven-point scale (1-not at all happy; 7-very happy) (Aknin et al., 2012). Previous research has confirmed the validity and reliability of such coding, as it shows that naïve coder ratings are correlated highly with Baby FACS, a validated measure of emotional coding (Oster, 2006; Aknin et al., 2012). One coder coded all the videos, and another one coded a random 25% of the sample. The inter-rater agreement between these two coders was high, *r* = 0.90. There were 3 phases in total: (1) *sharing* phase, which begins when the child started to put the stickers into either envelope (the recipient’s or the child’s), and ends when the child finished this process (e.g., s/he reported “I am done” or stood up and walked toward the experimenter). Children’s happiness was coded separately when putting stickers into their own envelope and the recipient’s (referred to as “*self*” and “*recipient*” phase below); (2) *Pre-sharing* phase: the 2-s interval before sharing took place; (3) *Post-sharing* phase: the 2-s interval after sharing had finished.

## Results

### Sharing Behavior

Data were analyzed using IBM SPSS 21.0. Our first analysis focused on children’s sharing behavior. Descriptive data are shown in **Table 1**. A preliminary analysis revealed no effect of gender so we collapsed data across boys and girls. We conducted a 2 (condition: altruistic sharing vs. obligated sharing) × 2 (age: 3 vs. 5) between-subject factor analysis, with the number of stickers shared overall as the dependent variable. As shown in **Table 1**, there was (1) a significant main effect of condition, *F*(1,135) = 11.05, *p* = 0.001, Δ*η*^2^ = 0.08, with children sharing more in the obligated sharing condition (*M* = 1.77, *SD* = 1.25) than in the autonomous sharing condition (*M* = 1.00, *SD* = 1.13); (2) a significant main effect of age, *F*(1,135) = 7.14, *p* = 0.008, Δ*η*^2^ = 0.05, with 5-year-old children (*M* = 1.59, *SD* = 1.19) sharing more than 3-year-olds (*M* = 1.02, *SD* = 1.27). The interactive effect of condition and age was not significant, *F*(1,135) = 1.444, *p* = 0.232, Δ*η*^2^ = 0.01.

**Table 1:** Means (standard deviation in parentheses) of children’s sharing behavior as a function of age and condition.

	Age 3		Age 5	
	Autonomous	Obligated	Autonomous	Obligated
Mean number of stickers shared overall^a^	0.81 (1.21)	1.25 (1.33)	1.12 (1.07)	2.04 (1.13)
Proportion of children who did not share at all	66.67%	37.5%	32.56%	13.33%
Proportion of children who shared stickers	33.33%	62.5%	67.44%	86.67%
Proportion of children who shared less than half	18.52%	45.83%	48.84%	40%
Proportion of children who shared half^b^	14.81%	16.67%	18.60%	46.67%
Mean number of stickers shared in children who shared^a^	2.44 (0.53)	2.00 (1.13)	1.66 (0.90)	2.36 (0.84)

^a^*The total number of stickers was 6; ^b^only one 3-year-old girl and one 5-year-old boy put more than half of the stickers (5 and 4 stickers, respectively) in the recipient’s envelope in the obligated sharing condition; these two children’s data were included into the category of “children who shared half.”*

In addition, Chi-square analyses on the number of children who shared (i.e., children who put at least 1 sticker into the recipient’s envelope) showed results consistent with those above (**Table 1**). The proportion of children who shared in the obligated sharing condition (54/69) was significantly higher than that in the autonomous sharing condition (38/70), χ^2^(1) = 8.93, *p* = 0.003. This result was found in both 3-year-olds [χ^2^(1) = 4.34, *p* = 0.037] and 5-year-olds [χ^2^(1) = 4.63, *p* = 0.031]. Moreover, there were significantly more 5-year-old children (68/88) who shared than 3-year-olds (24/51), χ^2^(1) = 13.17, *p* < 0.001.

Interestingly, a 2 (condition: altruistic sharing vs. obligated sharing) × 2 (age: 3 vs. 5) ANOVA test shows that there was a significant interactive effect of condition and age in the number of stickers shared in children who shared, *F*(1,88) = 6.99, *p* = 0.010, Δ*η*^2^ = 0.07. As shown in **Table 1**, for children who shared, 3-year-old children shared the same number of stickers in both conditions, *F*(1,88) = 1.40, *p* = 0.240, Δ*η*^2^ = 0.02, but 5-year-olds shared more stickers in the obligated sharing condition than in the autonomous sharing condition, *F*(1,88) = 10.40, *p* = 0.002, Δ*η*^2^ = 0.11. Moreover, Chi-square tests showed that in children who shared, the proportion of 5-year-old children who shared half of the stickers was higher in the obligated sharing condition than in the autonomous sharing condition, χ^2^(1) = 4.69, *p* = 0.030, but there were no significant differences in 3-year-olds, χ^2^(1) = 0.80, *p* = 0.412 (**Table 1**).

### Sharing Happiness

Pearson correlations showed that the correlations between the number of shared stickers and children’s happiness rated by the coder in each phase (i.e., pre-sharing, self, recipient, and post-sharing) in each condition was not significant, *r*s ranged from -0.14 to 0.08, *p*s > 0.10. No significant correlations were found between age (months) and happiness either, *r*s ranged from -0.06 to 0.12, *p*s > 0.10. ANOVA tests with age (3 years vs. 5 years) as the independent variable and children’s happiness in each phase as the dependent variable showed no significance effects either, *p*s > 0.10. These results suggest that children’s happiness in each phase did not vary according to how many stickers children shared, or how old they were.

For our primary question, we analyzed whether children’s happiness differed across conditions with ANOVA tests. Overall, there were no significant differences in children’s happiness during the pre-sharing phase, *F*(1,137) = 0.94, *p* = 0.335, Δ*η*^2^ = 0.007, nor during the post-sharing phase, *F*(1,137) = 0.005, *p* = 0.944, Δ*η*^2^ < 0.000. During the sharing process, only children who shared had data in the “recipient” phase (children who did not share did not put stickers into the recipient’s envelope, thus had no data). We first compared ‘generous’ and ‘selfish’ (children who did not share at all) children’s happiness across conditions. A 3 (phase: pre-sharing, self, post-sharing) × 2 (condition: altruistic sharing vs. obligated sharing) × 2 (‘generous’ vs. ‘selfish’) mixed-factor analyses showed a significant main effect of phase, *F*(2,134) = 29.38, *p* < 0.001, Δ*η*^2^ = 0.31. No other significant effects were found, *p*s > 0.10. *Post hoc* analyses showed that children were happier during the post-sharing phase (*M* = 4.66, *SD* = 0.10) than in the pre-sharing phase (*M* = 4.09, *SD* = 0.71) and “self” phase (*M* = 4.12, *SD* = 0.49), *p*s < 0.001. Therefore, all children were happier in the post-sharing phase (when they finished the task and were ready to return to their classroom with stickers) than in the pre-sharing phase (when they were asked to make a decision about sharing), regardless of which condition they were in.

**FIGURE 1:**
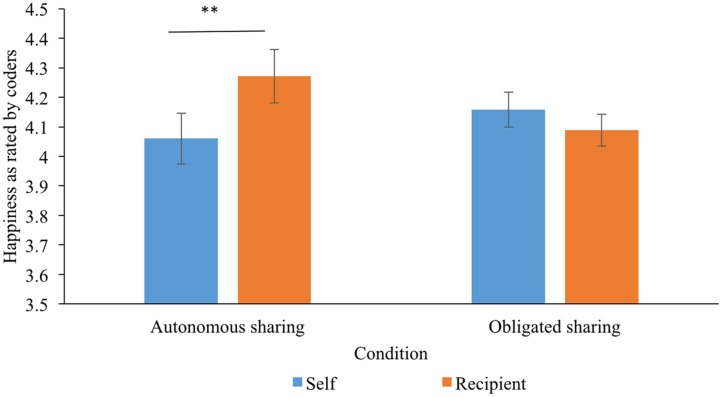
Generous children’s happiness rated by coders when putting stickers into their own and the recipient’s envelope in two conditions. Error bars represent the standard errors of the means. ^∗∗^*p* < 0.01.

We then compared ‘generous’ children’s happiness when putting stickers into their own envelope and the recipient’s envelope. For the 92 children who shared (*n* = 38, 54, respectively, for the autonomous and obligated sharing condition), we conducted a 2 (between-subject factor, condition: autonomous sharing vs. obligated sharing) × 2 (between-subject factor, age: 3 vs. 5) × 2 (within-subject factor, phase: self vs. recipient) mixed-factor analysis of variance. As shown in **Figure 1**, results revealed a significant interactive effect of condition and phase, *F*(1,88) = 5.85, *p* = 0.018, Δ*η*^2^ = 0.06. Further analysis on the interactive effect showed that in the autonomous sharing condition, children were happier when putting stickers into the recipient’s envelope (*M* = 4.27, *SD* = 0.56) than when putting stickers into their own envelope (*M* = 4.06, *SD* = 0.53), *p* = 0.007. By contrast, no significant differences between children’s happiness in the “self” (*M* = 4.16, *SD* = 0.44) and “recipient” phase (*M* = 4.09, *SD* = 0.39) were found in the obligated sharing condition, *p* = 0.205. In addition, children’s happiness when sharing with the recipient was marginally higher in the autonomous sharing condition (*M* = 4.27, *SD* = 0.56) than that in the obligated sharing condition (*M* = 4.09, *SD* = 0.39), *p* = 0.070, whereas their happiness when putting stickers into their own envelope did not differ significantly between the two conditions, *p* = 0.339. Moreover, there was no significant main effect of age [*F*(1,88) = 0.36, *p* = 0.549, Δ*η*^2^ = 0.004], nor any interactive effects involving age, *p*s > 0.10, which was consistent with the above correlational results that age was not significantly correlated with children’s happiness.

## Discussion

Previous studies have documented positive affective benefits of altruistic sharing (e.g., Dunn et al., 2008, 2014; Aknin et al., 2012, 2013a), but has left open a question whether the happiness gains exist when sharing is obligated as expected by social norms, in which case sharing is likely to be carried out due to pressure. The current study examined whether 3- and 5-year-old children exhibited as much happiness when sharing was obligated as when sharing was autonomous. There were three main findings: (1) overall children shared more items when sharing was obligated than autonomous, demonstrating their conformity to social norms; (2) for children who shared, 3-year-olds shared the same amount of stickers in both conditions, but 5-year-olds shared more stickers (and a larger proportion of 5-year-olds shared half) in the obligated sharing condition than in the autonomous sharing condition, suggesting that 5-year-olds followed the merit-based sharing norm more strictly than 3-year-olds did; (3) children who shared were happier when putting stickers into the recipient’s envelope than putting stickers into their own envelope in the autonomous sharing condition, but not in the obligated sharing condition.

The current study demonstrates that in a naturalistic situation, autonomous sharing rewards children with positive emotions in a real-time context. In our autonomous sharing condition, sharing was purely altruistic and voluntary, because the child was told that those stickers were rewarded to him or her, the recipient did not contribute to earning the reward, and the recipient failed to get his/her own reward due to the experimenter’s carelessness (she ran out stickers), thus it was not the child’s responsibility to share. When left alone to make their own decisions, children who decided to share exhibited greater happiness when putting stickers into the recipient’s envelope than putting stickers into their own envelope. The present study thus replicates previous research showing that altruistic giving to others makes people happy (e.g., Dunn et al., 2008, 2014; Aknin et al., 2013a), and further extends these Western studies of adults to preschoolers in Chinese populations. The finding is also significant because it shows that the happiness gains could not only be self-reported by participants as in previous studies (Dunn et al., 2008, 2014; Aknin et al., 2011a,b, 2013a,b, 2014), but could also be observable to third parties (see also Aknin et al., 2012, 2014). Moreover, the current study generalizes the previous finding on toddlers’ sharing and happiness to a more naturalistic situation; the prior study on toddlers’ affect used a hand puppet as a social partner, and compared toddlers’ happiness when they were asked to share their own treats versus when they were asked to share an experimenter’s treats (Aknin et al., 2012). Sharing was thus toward a hand puppet, and was suggested by an experimenter. Our study provides evidence that in a more realistic situation when children were led to believe that they were sharing with a peer, spontaneous sharing could benefit children with happiness.

Critically, the present study provides the first evidence that sharing under social norms may not benefit children with positive affect. In the obligated sharing condition, children knew that the recipient contributed to earning the reward, and they were told that the stickers were rewarded to both himself/herself and the recipient for completing the puzzle. Consistently, a larger proportion of children shared, and they also shared more stickers than in the autonomous sharing condition. This shows that both 3- and 5-year-old children could distinguish the two sharing contexts, and they conformed to the social norm of merit-based sharing. However, they were not happier when putting stickers into the recipient’s envelope than keeping stickers for themselves in the obligated sharing condition as seen in the autonomous sharing condition. These findings support the argument that positive mood is associated with autonomous sharing, but not with obligated sharing. Together with prior studies showing that happiness increases prosocial behaviors (Aknin et al., 2011a), our research may support the theoretical hypothesis that one proximate mechanism of prosocial behavior is via experiencing positive affect. Note that the role of socialization, empathy and sympathy toward the recipient who had no stickers cannot be completely ruled out in this situation of sharing. We are arguing that, in addition to these factors, children may find altruistic and voluntary sharing rewarding, which could help explain the puzzle why people engage in costly giving without obvious benefits. By contrast, the emotional reward can hardly explain children’s sharing behavior when it is expected by social norms, because when children follow the social norms to share, they do not benefit from positive affect.

These findings are consistent with previous studies on “pleasure-based prosocial motivation” and “pressure-based prosocial motivation” (e.g., Gebauer et al., 2008). According to Gebauer et al. (2008), a prosocial orientation can be motivated by the anticipation of positive affect (pleasure), or by fulfilling a duty or conforming to a social norm (pressure). The current study thus provides important evidence to distinguish between pleasure and pressure based prosocial motivation even in preschool children: when there was hardly any pressure to share, fewer children shared, but children who did share gained pleasure when giving resources to others; by contrast, when there was pressure to share, children shared more, but did not gain that much pleasure.

Notably, children did not show significant differences in displaying their happiness between the autonomous and obligated sharing condition in the pre-sharing and post-sharing phase, nor when keeping stickers for themselves. There were no age differences in children’s emotional expression either. In fact, all of them were the happiest in the post-sharing phase, when they were done with sharing and were ready to return to their classrooms with their stickers. The no significant differences between children’s happiness when putting stickers into the child’s own envelope versus the recipient’s envelope in the obligated sharing condition is worth future pursuit. It suggests that obligated sharing does not benefit children with happiness, but it does not lead to a dampening of positive affect either. One possibility is that children take it for granted to share in the obligated sharing condition, without much emotional change. Another possibility is that children were unhappy due to the pressure on one hand, but on the other hand, they are relaxed after following a social norm; the two opposite emotions washed out each other, resulting in no significant differences. Future studies incorporating participants’ self-report and biological measurements may provide insights into this issue.

One interesting question for future studies to address is how children behave and feel when allocating stickers if they have completed the puzzle task in the company of the other child, rather than simply hearing about the existence of the other child as shown in the current study. It is very common in developmental research that subjects are asked to divide actual resources between themselves and anonymous others (Blake and Rand, 2010; Gummerum et al., 2010; Engelmann et al., 2013; Xiong et al., 2016). This resource-allocation paradigm has many methodological advantages. For instance, it enables participants to make individual choices in a one-shot interaction, in which the allocator and the recipient are anonymous to each other; therefore, the concern of retaliation, reciprocation or reputation is minimized. Moreover, it is hard to control children’s behaviors in reality to ensure that they make equal contributions to completing the puzzle, as described in our study. In addition, if the recipient is present, s/he may request that the participant shares, and the verbal or non-verbal cues from the recipient will influence the participant’s decisions (Brownell et al., 2009; Wu and Su, 2014; Wu et al., under review). Because of these concerns, we used an anonymous situation and told children about the other child’s effort, instead of having children interact with a real partner. Children had to pass the comprehension questions before sharing, and they indeed shared more in the obligated sharing condition than in the autonomous sharing condition. These findings are consistent with studies in which children shared resources after collaborating with real social partners directly (Warneken et al., 2011), showing that our manipulation was overall successful. Nevertheless, this type of experimental situation might not represent real-life situations. In addition, we do not know how children feel during the process of sharing with a real partner after direct interactions, which requires future investigation.

Despite our interesting findings, there are a few limitations of the current study. The first limitation is that we could not fully rule out social pressure in the autonomous sharing condition, in which there was one envelope designated for the recipient, and the child would speculate that the number of stickers s/he shared would eventually be discovered by the experimenter or the recipient. We used envelopes to create a situation that children would feel free to share what they wanted because others could not find out whether they shared and how much they shared as obviously as sharing without envelopes. We also told participants that it was ok to put all stickers in one envelope. In fact, the sharing context was the same in both conditions, yet 66.67% of 3-year-olds and 32.56% of 5-year-olds did not share at all in the autonomous sharing condition, which was significantly higher than in the obligated sharing condition. This shows that, though there might be some pressure in the autonomous sharing condition, it was less than in the obligated sharing condition. The second limitation is that we created an obligated sharing condition by implementing a merit-based sharing norm, but there might be a social norm of fairness to follow in the autonomous sharing condition as well. Though this is possible, we think that for preschoolers, sharing equally after collaboration is a stricter rule to follow than simply being egalitarian with others. Previous studies have shown that children do not allocate resources in a fair manner until around 7–8 years old (Damon, 1975; Fehr et al., 2008; Rochat et al., 2009; McCrink et al., 2010; Sheskin et al., 2014), though infants in their second year already show an expectation for equal distribution when evaluating resource distribution to third-parties (Geraci and Surian, 2011; Schmidt and Sommerville, 2011; Sloane et al., 2012). Preschoolers tend to act in a self-interested way, preferring others to get less relative to themselves, which demonstrates a discrepancy between their knowledge of fairness norm and true behavior (Smith et al., 2013; Blake et al., 2014). By contrast, even 3-year-olds engage in merit-based sharing after collaboration (Warneken et al., 2011; Baumard et al., 2012; Kanngiesser and Warneken, 2012; Hamann et al., 2014; Schmidt et al., 2016). Consistent with previous studies, we also found that even 3-year-olds shared more in the obligated sharing condition than in the autonomous sharing condition; furthermore, very few children shared half of the stickers in the autonomous sharing condition, showing that they were not simply invoked by a concern for fairness. Therefore, for preschoolers, we argue that the pressure to share in the obligated sharing condition was more than in the autonomous sharing condition; however, future studies are necessary to replicate our findings by manipulating ‘pressure’ in a more clear and straightforward way.

In sum, this study enriches our understanding of the relation between generosity and happiness, and contributes to identifying the underlying mechanisms of young children’s sharing behavior. It extends previous studies on adults’ and toddlers’ prosocial behavior and positive mood by demonstrating that Chinese preschoolers gain affective benefits after spontaneous sharing in a naturalistic situation. In addition, it adds evidence to a potential psychological mechanism that leads to higher generosity via influencing positive feelings; more importantly, it suggests that affective benefits may depend on the motivation for sharing. Therefore, affective benefits and social norms may drive children’s prosocial behaviors separately: when sharing is altruistic and voluntary, children experience positive mood, which leads to further sharing subsequently (Aknin et al., 2011a); however, when sharing is expected by social norms, children are likely to follow the social norm and share even more than when sharing is autonomous, although they do not gain positive affect.

## Author Contributions

ZW and ZZ proposed the concept and designed the study. ZZ and RG collected and coded the data. ZW and ZZ analyzed the data. All authors made substantial contributions to the interpretation of data. ZW drafted the study, ZZ and JGL revised the manuscript for important intellectual content. All the authors approved the version to be published and agreed to be accountable for all aspects of the work in ensuring that questions related to the accuracy or integrity of any part of the work are appropriately investigated and resolved.

## Conflict of Interest Statement

The authors declare that the research was conducted in the absence of any commercial or financial relationships that could be construed as a potential conflict of interest.
